# Older adults are not more susceptible to acute muscle atrophy after immobilisation compared to younger adults: a systematic review

**DOI:** 10.1007/s00068-021-01694-0

**Published:** 2021-06-03

**Authors:** Harry Hodgson, Michael Wilkinson, Scott Bowen, Peter Giannoudis, Anthony Howard

**Affiliations:** 1 Leeds Teaching Hospitals NHS Trust, Leeds, UK; 2 University of Manchester NHS Foundation Trust, Manchester, UK; 3 University of Leeds, Leeds, UK; 4 University of Leeds, LIRRM, Leeds, UK; 5 LIRRM, Academic T&O Unit, Clarendon Wing, D floor, Great George Street, Leeds, LS1 3EX UK; 6 NDORMS, University of Oxford, Oxford, UK

**Keywords:** Muscle atrophy, Ageing, Immobilisation, Systematic review

## Abstract

**Purpose:**

To identify if older adults are more susceptible to acute muscle atrophy compared to younger adults.

**Methods:**

All studies whose design involved a period of enforced immobilisation and a comparison between an older (> 40) and a younger cohort (< 40) were included. Outcome of interest was change in muscle mass, measured by radiological techniques or histological analysis of fibre size. Medline, Embase and Cochrane databases were systematically searched and records screened by two independent reviewers. Studies selected for inclusion were critically appraised and individually assessed for risk of bias. GRADE framework guided the assessment of quality of studies.

**Results:**

Eight articles were included (193 participants). 14 (7.3%) were female and 102 (52.8%) were in older groups. Mean age for older adults was 66.3 years and for younger adults 23.3 years. Immobilisation periods spanned 4–14 days as simulated by bed rest, limb brace or limb cast. Studies measured muscle mass by DXA, CT, MRI or fibre cross-sectional area, or a combination of each. Muscles studied included quadriceps, adductor pollicis, vastus lateralis or combined lean leg mass.

Of the radiological measures, three studies (74 participants) reported greater atrophy in the older group, three studies (76 participants) reported greater atrophy in the younger group. Reduction in muscle mass varied in older adults between 0.19 and 0.76% per day, and for younger adults between 0.06 and 0.70% per day. Due to substantial heterogeneity, a meta-analysis was not performed. Five studies reported fibre size. Change in fibre size varied considerably between each study, with no convincing overall trend for either older or younger groups.

**Conclusion:**

The current literature suggests that there is no difference in the rate of muscle atrophy after immobilisation in older people compared to younger people, and therefore that older people are not more susceptible to atrophy in the acute setting. However, the findings are inconsistent and provide statistically significant but opposing results. There is a lack of high-quality research available on the topic, and there is a paucity of literature regarding atrophy rates in women.

## Introduction

Low skeletal muscle mass is well recognised as a cause of morbidity and is independently associated with functional impairment in older people [1]. Muscle atrophy has also been shown to predict survival in a number of disease states [2–4]. When associated with biological ageing, loss of muscle mass has been termed sarcopenia [5] and a significant body of work has been published investigating the consequences of loss of muscle mass in older adults [6].

Whilst the association between loss of muscle mass and age is well established, it has been proposed that multiple short periods of immobilisation in later life and the resulting acute atrophy contribute significantly to sarcopenia and poorer clinical outcomes [7]. Even short periods of hospital admission following elective hip arthroplasty have been associated with significant lower limb muscle loss in older patients [10]. Further, the rate of disuse atrophy decreases exponentially with the duration of disuse [7], and therefore, it is likely that strategies to combat muscle loss will be most successful in the initial period of immobilisation. A common cause of disuse atrophy is hospital admission for illness or injury, and length of stay averages between 5 and 10 days in the European Union [8]. This is particularly relevant to orthopaedic and trauma admissions, where treatment often involves deliberate immobilisation of affected limbs. Clinical opinion regarding post-fixation activity levels is divided, and there is a fine balance between early mobilisation and reduced atrophy, against the risk of early mobilisation resulting in catastrophic failure of fixation [9].

Population ageing is now a global phenomenon, and particularly marked in Europe, the United States and Japan [11]. The prevalence of sarcopenia is estimated at 10% in the over-60 s worldwide [12], and along with other age-related syndromes represents an increasingly important health consideration. This is particularly relevant to acute injury, where muscle atrophy may be both a cause [13] and a consequence of hospital admission.

Multiple strategies have been proposed in the treatment of sarcopenia, including androgens, growth hormone, vitamin D, and novel therapeutics targeting, for example, myostatin [14]. Protein or amino acid supplementation combined with resistance training has also shown some promise [15, 16].

Much of the literature regarding disuse atrophy has understandably focussed on older adults, where the burden of loss of independence is concentrated [17]. There is a relative paucity of data describing differences in the natural history of disuse atrophy between young and older adults. Such data may aid in the identification of novel targets for therapeutics and in the generalisation of results between age groups. In this review, we aim to summarise the available evidence comparing rates of acute muscle atrophy induced by immobilisation in older and young patients.

## Methods

The Preferred Reporting Items for Systematic Reviews and Meta-analyses (PRISMA) were used to guide the systematic review and presentation of results. PROSPERO registration number CRD42021226197. Eligibility was determined using the Population, Intervention, Comparison and Outcomes model. For inclusion, studies must have (a) compared a cohort of younger participants against an older cohort. It is though that muscle atrophy is age related but there is no evidence whether this is linear or progressive. A mid-point in adult life was adopted of 40 years of age, as it is known that after this point atrophy increases due to more sedentary activity [18]. (b) Participants must have undertaken a period of immobilisation. (c) Any method of measuring muscle volume or mass was included, including radiological methods and muscle fibre size. When DXA was used to measure atrophy, we used the lean leg mass rather than whole body mass, because this is more precise because it is not diluted by non-contractile lean mass. Clinical estimations of atrophy, such as grip strength, were not included, due to concerns, these measures show great variability in findings [19] and lack the precision for measuring muscle mass compared to radiological and histological methods [20]. (d) At least two measures of atrophy must have been reported (i.e. before and after immobilisation) to determine the percentage atrophy for each cohort; (e) Old and young participant groups must have been treated identically where possible; (f) Participants could be healthy volunteers or patients with injuries.

Only English language results were included. There was no cut-off for publication date. The search was restricted to include human data only. Grey literature was excluded, including conference papers, case reports and unpublished research. The electronic databases Ovid Medline, Ovid Embase, and Cochrane library were searched on 10/12/2020. The keywords searched were muscle atrophy, muscle wasting, older adults, young adults, bed rest and immobilise. Key words were expanded to include the following Medical Subject Headings (MeSH), including truncations (*): muscle atroph*, muscle fibre degeneration, muscle degeneration, muscle cell degeneration, muscle wast*, muscle recession, myoatrophy, myodegeneration, fatty infiltration, fatty degeneration, lean mass, bed rest, immobile*, trauma*, older adults, older patients, elderly, elders, geriatric, young, younger adults, younger patients, frail, senior, skeletal muscle. A manual review of bibliographies from relevant publications was also carried out for completion and to ensure the inclusion of relevant publications.

Citations were screened according to their title and abstract. Studies deemed to be potentially relevant were assessed according to the inclusion and exclusion criteria after reading the full text. Records were screened by two independent reviewers and any disagreements over inclusion were settled by consensus. Efforts were made to identify research using the same pool of patients and such publications were discarded to prevent participants being represented more than once.

Researchers (HH and MW) independently extracted the following information from all relevant studies: participant information including average age, gender, inclusion and exclusion criteria for each study, immobilisation intervention, whether controlled for dietary intake or not, whether there is any baseline difference in muscle mass, measure of atrophy, when atrophy was measured, percentage change in muscle mass for each group, significance between groups and whether any blinding took place at any level. The principal summary measure was the percentage change in the muscle mass from pre-immobilisation to post-immobilisation. Where a percentage change was not provided within a study, this was derived based on the data provided. Any conflicts in information collected were settled mutually with a third researcher (AH).

Studies selected for inclusion were critically appraised and individually assessed for risk of bias according to the authors’ judgement. The Grading of Recommendations, Assessment, Development and Evaluations framework (GRADE), a widely accepted tool for grading the quality of evidence, was used to assess the quality of studies based on the risk of bias, imprecision, inconsistency, indirectness and publication bias for each study [21]. Statistical heterogeneity between study results will be considered.

## Results

**Fig. 1:**
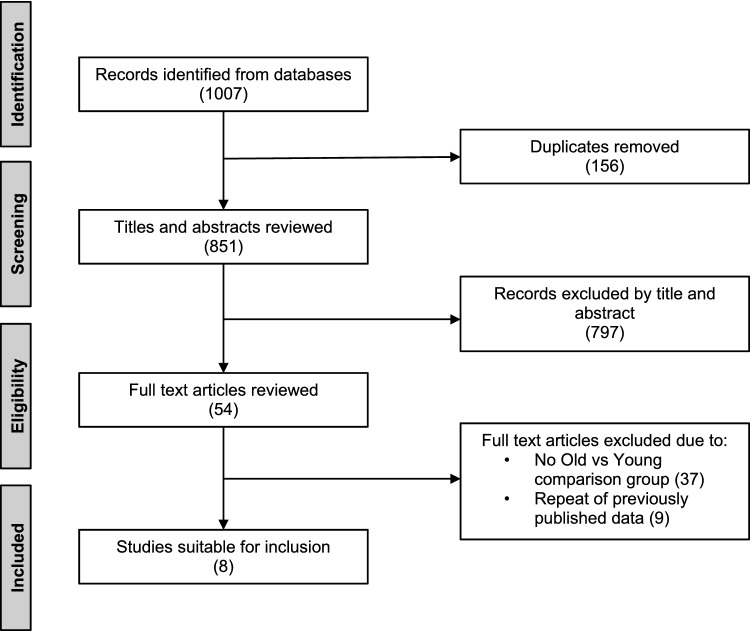
Flow diagram of study selection

Searching revealed 1007 records. After removing duplicates, 851 articles were screened for further analysis. Records were excluded based on assessment of suitability from title and abstract. 54 full text articles were assessed for inclusion and exclusion criteria. Studies were excluded for not having two cohorts comparing young participants against older participants. Nine studies repeated previously published data and were excluded. Eight studies met the inclusion and exclusion criteria [22–29]. Figure 1 displays a flow diagram of study selection.

In total, 193 (14; 7.3% females) participants were included; 102 (52.8%) were older adults and 91 (47.2%) younger adults. Only one study included female participants [25]. The mean age for older adults was 66.3 years and younger adults 23.3 years. One study had a 4 day immobilisation period [28], two had a 5-day immobilisation period [25, 27], and five studies had a 14 day immobilisation period [22–24, 26, 29, 30]. One study blinded a part of the study by blinding the radiologist [23].

Six studies measured atrophy by radiological methods, listed in Table 1. Three studies used MRI to determine muscle volume [22–24]; three studies used DXA to determine lean leg mass [25–27], one of which also used CT to determine quadriceps muscle volume [27]. One of the DXA studies [25] used MRI also, but only for some of the patients, so the MRI data were not included. Five studies assessed either the quadriceps [22, 23], the overall leg mass [25, 26] or both [27]. One study looked at a muscle in the upper limb, the adductor pollicis [24].

**Table 1:** Table of citations: radiological measures of atrophy

Article author and year	Sample size (*n*)	Older adult (*n*)	Young adult (*n*)	Older adult age (years)	Young adult age (years)	Female (*n*)	Immobilisation period (days)	Intervention type	Atrophy measure (unit of measure)	Change old (% from baseline)	Change young (% from baseline)	Rate of change old (%/day)	Rate of change young (%/day)	Mean difference in rate of change between young and old (%/day ± SD)	Significance young vs old (*p* value)	GRADE score [21]
Reidy et al. (2018) [25]	23	9	14	66 ± 1.09	23.14 ± 0.84	14	5	Bed rest	DXA leg lean mass (kg)	− 3.82 ± 0.74	− 0.30 ± 0.67	− 0.764	− 0.06	− 0.704 ± 0.0839	0.012	Very low
Vigelso et al. (2015) [31]	32	15	17	68 ± 1	23 ± 1	0	14	Knee brace	DXA leg lean mass (kg)	− 1.1 ± 3.2% (derived)	− 5.6 ± 2.8 (derived)	− 0.188	− 0.406	0.218 ± 0.0286	Not reported	Very low
Pisot et al. (2016) [22]	23	16	7	59.6 ± 3.4	23.1 ± 2.9	0	14	Bed rest	MRI quadriceps muscle volume (cm^3^)	− 8.4 ± 3.7	− 5.7 ± 3.9	− 0.599	− 0.406	− 0.192 ± 0.0508	< 0.05	Very low
Wall et al. (2014)	24	12	12	70 ± 1	23 ± 1	0	5	Immobilised single leg	(1) CT quadriceps CSA (cm^2^)	− 1.5 ± 0.7	− 3.5 ± 0.5	− 0.3	− 0.7	0.4 ± 0.0689	< 0.001	Very low
									(2) DXA lean leg mass (kg)	− 1.1 ± 1.0	− 1.4 ± 0.7	− 0.22	− 0.28	0.06 ± 0.244	< 0.05	
Suetta et al. (2009) [23]	20	9	11	67.3 (range 61–74)	24.4 (range 21–27)	0	14	Whole leg cast	MRI quadriceps volume (cm^3^)	− 5.2 (No SD given)	− 8.9	− 0.371	− 0.636	0.264 ± 0.0371	< 0.05	Very low
Urso et al. (2005) [24]	28	20	8	67 ± 4	21 ± 2	0	14	Hand brace	MRI adductor pollicis muscle volume (cm^3^)	− 9.5	− 4	− 0.679	− 0.286	− 0.393	Not reported	Very low

Five studies measured atrophy directly by collecting a muscle biopsy and measuring fibre size by histological methods, as listed in Table 2. Three studies also used radiological measures of atrophy [22, 25, 31]; some papers reported this data in the same paper as reporting radiological findings, whilst others published it separately. In one study [22], the fibre size analysis is discussed in depth in a paper published later by the authors [32]. One paper measures fibre size [31] and later publishes the radiological measures of atrophy [26], but the patients are the same. All five studies [25, 28, 29, 31, 32] collected a biopsy from the vastus lateralis, and measured fibre size by cross-sectional area (CSA) via histological methods.

**Table 2:** Table of citations: measuring atrophy by fibre size immunohistochemical staining (IHC)

Article (author and year)	Sample size (*n*)	Older adult (*n*)	Young adult (*n*)	Older adult age (years)	Young adult age (years)	Female (*n*)	Immobilisation period (days)	Intervention type	Atrophy measure and muscle biopsied	Staining type	GRADE score [21]
Rejc et al. (2018) [32] (n.b. same cohort as Pisot et al. 2016 [22])	23	16	7	59.6 ± 3.4	23.1 ± 2.9	0	14	Bed rest	Myofiber CSA—*vastus lateralis*	IHC	Very low
Reidy et al. (2018) [25]	23	9	14	66 ± 1.09	23.14 ± 0.84	14	5	Bed rest	Myofiber CSA—*vastus lateralis*	IHC	Very low
Vigelso et al. (2015) [31] (n.b. same cohort as Vigelso et al. 2016 [26])	32	15	17	68 ± 1	23 ± 1	0	14	Knee brace	Myofiber CSA—*vastus lateralis*	Myosin ATPase and IHC	Very low
Suetta et al. (2012) [28]	23	12	11	66.8 (range 60–72)	24.3 (range 21–30)	0	4	Knee brace	Myofiber CSA—*vastus lateralis*	Myofibrillar ATPase	Very low
Hvid et al. (2010) [29]	20	9	11	67.3 ± 1.3	24.4 ± 0.5	0	14	Lower limb cast	Myofiber CSA—*vastus lateralis*	Myofibrillar ATPase	Very low

**Fig. 2:**
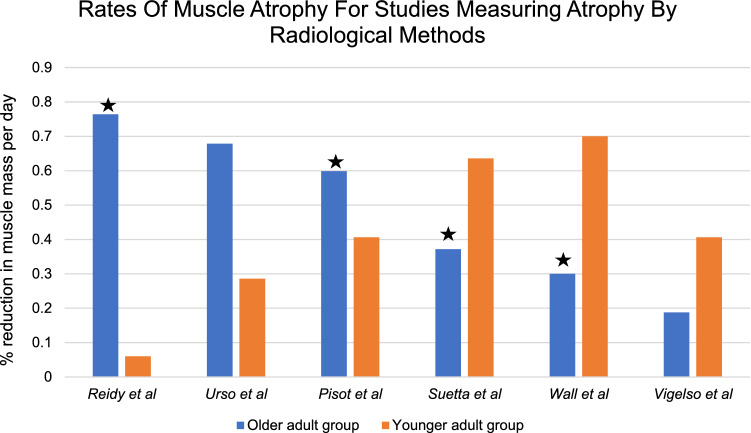
The daily percentage reduction in muscle mass for radiological measures of atrophy Wall et al. shows CT findings only. Star denotes *p* < 0.05 vs. young

**Fig. 3:**
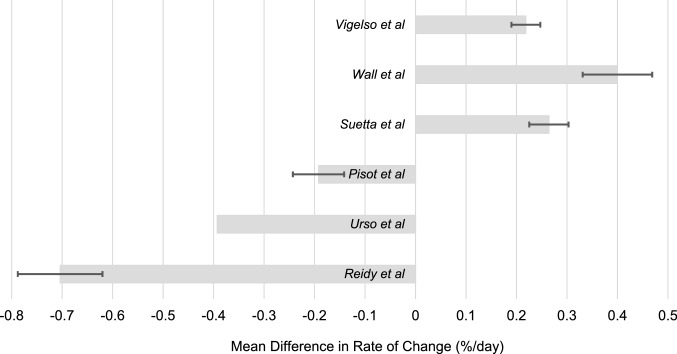
Mean difference in rate change in muscle atrophy for young vs. old groups

The mean rate of change for old and young groups was calculated for each study using radiological methods of measurement (percentage change in muscle mass or volume/immobilisation period in days) and is displayed in Fig. 2. Two measured atrophy after five days and both found significant differences in the change in muscle mass between old and young groups (Reidy et al.: Old = − 3.82% Young = − 0.3% *p* = 0.012, Wall et al.: Old = − 1.5% Young = − 3.5%, *p* < 0.05); one study found significantly greater atrophy in the older group, and the other study found significantly greater atrophy more in the younger group. Wall et al., the only study to use two measures, found a similar pattern for CT and DXA data, although a more profound change for the old group when measured by CT. Reidy et al. [25] used MRI in some of their patients as a late amendment to their protocol, which were also consistent with their DXA findings. Four studies measured atrophy after 14 days; two found a significant difference between old and young groups (Pisot et al.: Old = − 8.4% Young = − 5.7 *p* < 0.05, Suetta et al.: Old = − 5.2% Young = − 8.9% *p* < 0.05*)* whilst two did not report the significance, but still found variation between total mass lost in old and young groups (Vigelso et al.: Old = − 1.1%, Young = − 5.6%, Urso et al.: Old = − 9.5%, Young = − 4%. Two studies found greater change in muscle mass in the older group and two studies found greater change in muscle mass in the younger group. Mean difference in rate of change between young and old groups is shown in Fig. 3, with error bars representing standard deviation. Bars with negative mean difference show older adults atrophying at a greater rate, whilst positive mean difference shows younger adults atrophying quicker.

To assess consistency between studies using radiological measures, the Higgins *I*^2^ index was 85%; this suggests there is substantial heterogeneity. For this reason, it was deemed inappropriate to carry out a meta-analysis.

The studies that measured fibre size are discussed in turn due to differences in reporting between studies. Rejc et al. found a significant reduction in slow fibre CSA in the old group (19%), and a significant reduction of fast 2A fibre CSA in the young group (28%) [32]. Reidy et al. found that older adults fibre CSA reduced by 10.02%, but that younger adults fibre CSA increased by 8.42%; the authors speculated that a lack of sensitivity in measuring fibre CSA may be responsible for the findings [25].

Vigelso et al. reported that fibre CSA decreased in both groups [31], but did not publish the numbers. They also report that they found a decrease in type II fibre size in the younger group only and did not find a change in type 1 fibre size. Suetta et al. [28] included two separate populations in this study (the 14 day immobilisation arm is discussed separately [29]), and was the only study to include additional biopsies to the pre and post-immobilisation biopsies (at one and two days also). They found significant reductions in type I fibre CSA in both old (7.1%) and younger (8.1%) groups, and also significant reductions in type II fibre CSA in both old (10.9%) and young (12.6%). Hvid et al. found a significant reduction in young male CSA for type 1, type IIa and type IIx fibres (15–30%) after 14 days of leg immobilisation. They found that only type IIx fibre CSA decreased significantly (13.2%) in the older group. Not all of the subjects were included in the analysis due to a low quality of collected samples [29].

## Discussion

Whilst there is a wealth of information relating to sarcopenia in the elderly from chronic disuse, it has been unclear and controversial as to whether older adults atrophy at a different rate or are more susceptible to atrophy in the acute setting [33]. Within the orthopaedic discipline, clinical opinion over whether to permit or restrict post-operative early weight bearing is divided [9]. It is unclear whether certain age groups are more suited to a particular method, and as the choice is influenced by concerns over acute muscle atrophy, understanding whether older adults are more susceptible in the acute setting would improve decision-making. This is the first systematic review of the literature looking at age-related differences in acute muscle atrophy following immobilisation.

From the studies that used radiological methods to measure atrophy, three studies reported greater atrophy in the older group [22, 24, 25], and three studies reported greater atrophy in the younger group [23, 26, 27]. There is huge variability between studies, and four studies found significantly differences between old and young groups, although two for greater atrophy in the younger group and two for greater atrophy in the older group. These findings are diametrical apposed, and it is difficult to meaningfully interpret statistically significant opposing results. Furthermore, it would be expected at least some of the studies would have found similar rates of atrophy between old and younger groups with no significant difference between change in muscle mass.

Interestingly, there are inconsistencies in the results. For example, the study [25] that found the greatest daily rate of decline in all of the older groups found the smallest rate of decline in all of the younger groups. It is known that muscles atrophy at different rates, and atrophy is more profound in the weight bearing muscles in comparison to the upper limbs and trunk [34]; therefore, it is surprising that the only study that measured atrophy in a non-weight bearing limb also demonstrated the most profound atrophy in the younger group [23]. In the lower limb, atrophy is more profound in the ankle-plantar flexors, calves and knee extensors compared to knee flexors or gluteal muscles [35–37]. Based on this, it would be expected to observe more extensive atrophy from the studies looking at quadriceps muscle volume only by MRI, in comparison to whole leg lean mass on DXA. From the studies that measured atrophy by fibre size, it is difficult to compare results due to a lack of consistency over reporting the changes in specific fibres, with some choosing to group all fibres and some not reporting the numbers at all. Similar to when atrophy is measured by radiological measures, there is substantial variability in the results, including unusual and unexplained findings such as an increase in fibre size in younger adults in one study [25].

The evidence for no difference between groups is weak due to the inconsistencies in the results between studies. Variability in findings may be explained by the limited research available; just six studies using radiological measures and two additional using fibre size were suitable for inclusion, all of which include between 20 and 32 participants. Only one study blinded a part of the study by blinding the radiologist interpreting the scans [23]; the remaining radiological studies are at risk of radiologist confirmation bias. Some studies do make attempts to control for confounding variables such as oral intake [22, 25, 27] and by considering baseline muscle mass [22, 23, 27].

Additionally, measuring atrophy is difficult. There are a variety of investigations, including radiological scans, bioelectric impedance [38], and measuring fibre size [33]. Each investigation has limitations, and there is a lack of information on the accuracy of each method [20]. DXA is recognised as the gold standard in clinical practice [20], but this is largely due to the downsides associated with CT and MRI in clinical practice, specifically radiation exposure and high cost of operation, rather than because it is the most accurate measure of skeletal muscle atrophy. MRI is recognised as more appropriate for small scale research studies like the ones in this review, for precise measures of muscle volume [20]. However, including different methods does not explain the variation in findings alone, and there is no obvious relationship between all the studies using MRI or DXA, albeit this is only three studies for each method. Measuring atrophy by fibre size is also challenging. Muscle atrophy involves both fibre atrophy and fibre loss, and it is controversial as to the contribution of each of these to the overall whole muscle atrophy [33]. Another difficulty is that atrophy is fibre-type dependent, meaning different types of fibre can atrophy at different rates between the two groups. With such variability between studies, this may also mask the effect of studies which have only included analysis of overall average fibre size rather than of the different types of fibre. This adds another complexity when comparing the findings between studies.

The lack of a significant relationship between age and susceptibility to acute muscle atrophy after a period of immobilisation is relevant to clinical practice. Within orthopaedic surgery, post-operative rehabilitation protocols may not need to differentiate between older and younger patients, and age should not guide decision-making alone. Additionally, the similarities between older and younger groups in the response to immobilisation may mean future research in young patients is equally applicable to older patients. Whilst beyond the scope of this review, there do appear to be differences in the rate of recovery of atrophied muscle after short-term immobilisation [33]. Older adults recover muscle more slowly [30], and are less able to recover atrophied muscle [39]. Understanding and explaining these differences would be a beneficial future research focus for reducing the burden of acute muscle atrophy on older patients.

### Strengths and limitations

There are several limitations to this systematic review. The nature of the topic makes it complicated to analyse, and small study sizes makes age-related differences between groups complex. The short length of follow-up raises questions about whether atrophy is more convincing in older populations if given more time to develop. Another limitation is that the results may not be representative of older adults; the mean age for older groups is 66 years, relatively young in terms of today’s ageing population. Most studies excluded participants with co-morbidities; whilst this pragmatic approach makes it easier to compare participants, the ‘healthy’ population of older adults is unlikely to represent the actual older population for whom co-morbidities are commonplace. It is difficult to see a reasonable solution to this problem as it is not ethical to immobilise weaker people. Additionally, only 7.3% of participants were female, from one study. Any sex-specific differences are therefore uncertain, which is particularly concerning as women are overrepresented in certain disease states which predispose to disuse atrophy, such as in the hip fractures where the overall prevalence in over 50’s is more than double in women compared to men [40].

Three studies used bed rest as the immobilisation intervention, and the remaining studies used a brace or cast to immobilise a muscle or limb. The type of intervention does not appear to explain the variability in results, as there is no consistency between studies using bed rest compared to those using an immobilised limb. Also, the methods of artificial bed rest and immobilising a limb in healthy volunteers are unlikely to be representative of real world causes of immobilisation, such as acute illness or injury. When considering orthopaedic patients who frequently are subjected to bed rest or an immobilised limb following an injury, there is a lack of published data which measures age-related differences in acute atrophy; however, it has been observed that older persons have higher rate atrophy of at three months and 12 months following hip fracture compared to younger persons [41].

Another limitation that we found was the difficulty in collecting all of the data required from each study which may have allowed for further statistical analysis, particularly in the studies that measure fibre size. Also, this review only includes studies which include a comparator young group, because the primary aim was to analyse studies which have an identical immobilisation procedure between older and younger groups to ensure a meaningful comparison against a younger population. On review of these single group studies, the results do not change the findings in this systematic review.

## Conclusion

There is no significant relationship between rates of acute muscle atrophy and chronological age following a period of imposed immobilisation. As population ageing increases, the anecdotal philosophy that older adults are more susceptible to acute muscle atrophy is incorrect and should not guide decision-making in clinical practice. However, there is a lack of high-quality research on topic in general, particularly research demonstrating sex-specific effects, and in patients immobilised due to injury or disease. Further studies are desirable to allow better understanding of the natural history of short-term atrophy, which may pave the way for interventions which can reduce disuse atrophy in patients.

## Data Availability

Systematic review protocol available at PROSPERO CRD42021226197.
